# YTHDF3 suppresses interferon-stimulated gene (ISG)-dependent antitumor immunity and promotes HPV carcinogenesis in cervical cancer

**DOI:** 10.1038/s41419-025-08188-6

**Published:** 2025-12-26

**Authors:** Li Li, Dongmei Lin, Keyi Ao, Sheng Zhong, Hui He, Xin Li, Yi Hao, Xia Guo

**Affiliations:** 1 Shenzhen Key Laboratory of Viral Oncology; Department of Science and Innovation, Shenzhen Hospital, Southern Medical University, Shenzhen, PR China; 2 Shenzhen School of Clinical Medicine, Southern Medical University, Shenzhen, PR China; 3 Department of Medical Ultrasonics, South China Hospital, Medical School, Shenzhen University, Shenzhen, PR China; 4 Department of Pathology, Shenzhen Hospital, The University of Hong Kong, Shenzhen, PR China

**Keywords:** Cervical cancer, Tumour immunology

## Abstract

Interferon-stimulated genes (ISGs) serve as evolutionarily conserved mediators of antiviral defense and tumor surveillance. Emerging evidence underscores the non-oncogenic addiction of high-risk human papillomavirus (hrHPV) E6/E7 oncoproteins in maintaining malignant phenotypes and cervical carcinogenesis. Here, we leveraged CRISPR/Cas9-engineered YTHDF3-knockout (YTHDF3^−/−^) SiHa cells and *Ythdf3*^*−/*−^ mice to dissect the molecular arbiters governing m^6^A-dependent RNA regulation in HPV-driven carcinogenesis. To further elucidate the role of YTHDF3 in HPV-induced immunosuppressive tumor microenvironment (ITME) formation, we demonstrated that YTHDF3, an m^6^A RNA reader, suppresses type I ISGs responses. Notably, elevated m^6^A modification and YTHDF3 protein levels were observed in HPV^+^ CCa tissues. Mechanistically, YTHDF3 bound to the m^6^A methylation site of STAT3 mRNA, enhancing its stability and transcription efficiency. This YTHDF3-STAT3 axis repressed ISG (e.g., IRF7) transcription and IFN-α production, thereby compromising antiviral immunity and facilitating HPV E6/E7 persistence. Correspondingly, *Ythdf3*^*−*^ mice bearing TC-1 xenografts exhibited a significant reduction in immunosuppressive immune cell infiltration, including Tregs, M2 macrophages, and MDSCs, accompanied by enhanced CD8^+^ T cell activation. Collectively, our findings unveiled that YTHDF3-mediated upregulation of STAT3 suppresses the type I ISG expression, thus promoting HPV carcinogenesis and establishing an ITME. Taken together, our results suggest that targeting the YTHDF3/STAT3/IRF7 axis could be a promising therapeutic strategy against HPV-associated malignancies.

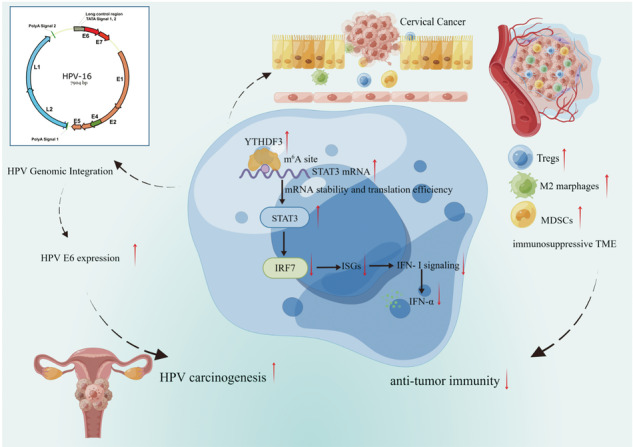

## Introduction

Interferon-stimulated genes (ISGs) function as evolutionarily conserved sentinels of antiviral defense and tumor surveillance, orchestrating cytostatic/cytotoxic responses and immunostimulatory cascades through type I interferon (IFN-I) signaling [1–3].

N6-methyladenosine (m^6^A), the most abundant type of mRNA modification, governs RNA metabolism (processing, splicing, translation, and decay) and is implicated in oncogenic reprogramming and immune modulation across diverse pathologies [4]. Cao and colleagues demonstrated that YTHDF3, an m^6^A reader protein, exerts its regulatory effects on mRNA stability and enhances mRNA translation efficiency [5]. Notably, it suppresses interferon-dependent antiviral responses by promoting FOXO3 translation [5, 6]. Given the integral role of interferons in antitumor immunity, understanding the impact of YTHDF3 on this pathway is crucial to elucidate its potential role in cancer immunology.

By infecting cutaneous and mucosal epithelial tissues, human papillomavirus (HPV), a double-stranded DNA virus, is the major culprit in HPV-associated diseases such as cervical cancer (CCa) and head and neck cancers [7]. The integration of the HPV DNA into the host genome during cancer progression leads to persistent expression of the E6 and E7 oncoproteins, which drive oncogenesis by disrupting the tumor suppressors p53 and pRb, respectively [8]. Their actions disrupt apoptotic pathway activation and stimulate cell proliferation, ultimately contributing to the progression of HPV-related malignancies.

In our previous research, we observed a marked increase in YTHDF3 expression in cervical cancer, promoting lymph node metastasis by reprogramming fatty acid metabolism [9]. However, the molecular mechanisms underlying YTHDF3’s role in HPV-driven carcinogenesis and immune escape remain unclear. Here, we demonstrate that YTHDF3 functions as a negative regulator of ISG expression by stabilizing STAT3 mRNA via m^6^A-dependent mechanisms. This YTHDF3-STAT3 axis suppresses IFN-α production and IRF7 activation, creating an immunosuppressive tumor microenvironment (ITME) conducive to HPV persistence. Collectively, our findings unveil a novel immune evasion axis in HPV-associated malignancies, proposing YTHDF3 inhibition as a rationale-based therapeutic strategy to restore antiviral immunity.

## Materials and methods

### Cell line and cell culture

The SiHa and CaSki cell lines were obtained from the Cell Bank of the Chinese Academy of Sciences (Shanghai, China). The TC-1 cell line was kindly provided by Dr. Lu (Shenzhen Bay Laboratory, China). All three cell lines were recently authenticated by STR profiling and were cultured in their respective media: SiHa and CaSki cells were cultured in RPMI 1640 medium (GIBCO, USA) supplemented with 10% FBS (Excell, Uruguay), 100 µg/mL penicillin, and 100 µg/mL streptomycin (BI, USA), and the TC-1 cell line was cultured in DMEM (GIBCO, USA) supplemented with 10% fetal bovine serum (Excell, Uruguay), 100 µg/mL penicillin, and 100 µg/mL streptomycin (BI, USA). All cultures were maintained in a humidified incubator at 37 °C and 5% CO_2_. In addition, the *YTHDF3* knockout SiHa cell line (YTHDF3^−/−^ SiHa) was generated via CRISPR/Cas9 technology from Haixing Biosciences (Guangzhou, China). The specific sg-YTHDF3-RNAs (JiKai, Shanghai) used are listed in Supplementary Table S2.

### Tissue microarray

This research analyzed 73 clinical samples, including 62 CCa samples and 11 normal cervical epithelium samples. Patient information associated with these samples was collected by Shanghai OUTDOB Biotech Co. This study was granted ethical approval by the Ethics Committee of Shanghai Outdo Biotech Company(No. SHYJS-CP-1801011).

### Plasmids

For functional studies, expression plasmids encoding wild-type YTHDF3 (YTHDF3-FLAG) and mutant YTHDF3 (YTHDF3-mut: residues 441–442 and 425–427) were generated. YTHDF3-FLAG and YTHDF3-mut expression plasmids were created by cloning YTHDF3 with a C-terminal FLAG tag into the pcDNA3.1 vector (HeYuan, China). The STAT3 overexpression plasmid was obtained from Gene JiKai (Shanghai, China). We utilized a plasmid containing the full-length HPV genome carrying DsRed (HPV-DsRed) from JiKai (Shanghai, China).

### siRNAs and cell transfection

To reduce targeted gene expression, commercially available siRNAs targeting STAT3 and negative controls were obtained from Gene Jima (Suzhou, China). Briefly, CCa cells were seeded into 6-well plates at a density of 2 × 10^5^ cells per well. Following overnight incubation, the cells were transfected with the appropriate siRNAs via the Lipofectamine 3000 reagent (Invitrogen, USA) according to the manufacturer’s instructions. Forty-eight to seventy-two hours posttransfection, the cells were harvested for subsequent protein or RNA extraction. The target sequences for STAT3 are listed in Supplementary Table S2.

### Cell transfection and lentiviral infection

Short hairpin RNAs targeting YTHDF3 (shYTHDF3) and nontargeting control (NC) were purchased from Jikai Gene (Shanghai, China) and used to achieve the knockdown of YTHDF3 expression in the cells. Cells were transfected with the shRNAs and incubated for 16 h at 37 °C. Following incubation, the viral medium was discarded and replaced with fresh culture medium. To select stably transfected cells, they were then treated with puromycin (2 µg/mL) for one week. This selection process resulted in the generation of stably silenced YTHDF3 cell lines.

### RNA extraction and quantitative RT‒PCR analysis

Total RNA was extracted from cells and tissues via TRIzol (Invitrogen, USA) according to the manufacturer’s instructions. The RNA (1 µg) was reverse transcribed into cDNA via a reverse transcription kit (YEASEN, China). cDNA and primers were synthesized with SYBR Green Master Mix (YEASEN, China) via an ABI 7500 RT‒PCR system (Applied Biosystems, Foster City, CA, USA). The PCR amplification conditions were as follows: 40 cycles of 95 °C for 30 s, 95 °C for 2 min, and 60 °C for 32 s. The relative mRNA expression levels were calculated via the 2^-ΔΔCt^ method with normalization to human GAPDH. All experiments were performed independently 3 times unless otherwise stated. The primers used for PCR analysis are listed in Supplementary Table S2.

### RIP sequencing

SiHa cells and YTHDF3^−/−^ SiHa cells were subjected to a series of procedures to identify YTHDF3-associated RNA targets. These procedures included ultraviolet (UV) crosslinking, cell lysis, immunoprecipitation using magnetic beads, isolation of RNA‒protein complexes, library construction, next-generation sequencing (NGS) on an Illumina NovaSeq 6000 platform, and bioinformatics analysis. The EpiTM mini long RNA-seq kit and EpiTM DNA Clean Beads were used for library preparation. Each group was sequenced in duplicate via 150-bp paired-end (PE150) sequencing.

### Ribo sequencing

SiHa cells and YTHDF3^−/−^ SiHa cells were subjected to a series of procedures, including cell lysis, flash-freezing, nuclease digestion, polysome fractionation, RNA fragment selection, library construction, on-machine sequencing, and bioinformatics analysis. cDNA libraries were generated via the QIAseq miRNA Library Kit (QIAGEN, catalog #1103679). Adapter sequences were subsequently removed from the raw sequencing data via Cutadapt software.

### RNA sequencing

Total RNA was extracted from SiHa and YTHDF3^−^ SiHa cells via TRIzol Reagent (Invitrogen, USA). The VAHTS Stranded mRNA-seq Library Prep Kit for Illumina V2 (Vazyme Biotech, NR612-02) was subsequently used for library preparation. The reads were aligned to the human reference genome, Ensembl GRCh38. FeatureCounts (v1.6.3) was used to quantify the number of reads mapped to the genome. Each group was sequenced in duplicate.

### Immunofluorescence (IF)

The cells were plated in six-well plates and allowed to achieve approximately 70% confluence. These cells were then transfected with a plasmid expressing the DsRed-tagged HPV genome (HPV-DsRed) via the Lipofectamine 8000 reagent (Beyotime, China). Forty-eight hours after transfection, samples were collected and fixed with 4% paraformaldehyde. The cells were then permeabilized with 0.5% Triton X-100 and blocked with 10% goat serum. Next, the samples were incubated overnight at 4 °C with either YTHDF3 or STAT3 antibodies. Following incubation, the samples were incubated with an Alexa Fluor 488-conjugated goat anti-rabbit IgG (H + L) secondary antibody. Nuclei were stained with DAPI, and the samples were imaged via a fluorescence microscope.

### MeRIP sequencing and MeRIP-qPCR

Total RNA was extracted from cells via TRIzol reagent (Invitrogen, USA). MeRIP sequencing was performed by Cloud See Biotech (China). Briefly, the isolated mRNA was fragmented, followed by immunoprecipitation with a m^6^A antibody (Abcam, USA). Reads mapped to the genome were then quantified via featureCounts (v1.6.3). Differential gene expression analysis was conducted via R software, with a false discovery rate (FDR) cutoff of 0.05. For MeRIP qPCR assays, total RNA (300 µg) was extracted from cells via TRIzol (Invitrogen, USA). The RNA was then fragmented and immunoprecipitated via the Magna MeRIP m^6^A Kit (#17--10499, Millipore). The enrichment of m^6^A on STAT3 was assessed via qPCR with specific primers. The data were normalized to the input RNA.

### mRNA stability assays and protein stability assays

To examine mRNA stability, SiHa and YTHDF3^−/−^ SiHa cells were treated with 4 µg/mL actinomycin D (Sigma, USA) for various durations. The cells were harvested at 0, 2, and 4 h posttreatment. The decay rate of each transcript was then calculated via the exponential functions of half-life analysis. To evaluate protein stability, SiHa and YTHDF3^−/^^−^ SiHa cells were treated with 200 µg/mL cycloheximide (CHX) for different durations. The cells were harvested at 0, 12, and 24 h posttreatment. The protein expression of STAT3 was then determined by western blot analysis.

### Mice

*Ythdf3*^−/−^ mice were generated on a C57BL/6 J background via CRISPR-Cas9 technology. All the mice were bred and maintained under specific pathogen-free conditions. To establish a congenic colony with minimal genetic variation, founder mice carrying the mutant alleles were backcrossed to C57BL/6 J mice for two generations. The mice were subsequently maintained by crossing heterozygous *Ythdf3*^−/−^ mice to ensure genetic homogeneity. The primers used for genotyping *Ythdf3*^*−/−*^sg1 mice were GTCACAAATAGTTACTTGAAGGTGG. All experiments involving animals were conducted according to the ethical policies and procedures approved by the Ethics Committee of the Shenzhen Hospital of Southern Medical University (No. 2022-0274).

### Xenograft models

Eight-week-old wild-type and *Ythdf3*^*-/-*^ C57BL/6 J mice (18–20 g) were raised under SPF conditions at the Southern Medical University Animal Center for the xenograft model. Genotyping and maintenance procedures were performed as previously described. All the mice were randomized into groups (*n* = 5 per group) and subcutaneously injected with luciferase-expressing TC-1 cells (1 × 10^7^ cells per mouse). After 10 days, the mice were sacrificed, and the tumors were dissected via the use of HPV titers, IHC, and flow cytometry. Blinding was not utilized in this study due to limited personnel availability for independent blinded assessments. To address potential bias, animals were randomly allocated to experimental groups, and all procedures followed standardized protocols with quantitative endpoints to minimize subjective interpretation. Raw data were independently verified by two investigators.

### Immunohistochemistry (IHC)

Clinical samples were processed via a PV-6000 kit (Zhongshan Jinqiao, Beijing) according to the manufacturer’s instructions. First, the paraffin-embedded tissues were dewaxed, and the antigens were retrieved at 120 °C for 10 min. Appropriate types of primary antibodies were used in the corresponding pH unmasking solutions. To block endogenous peroxide activity, the samples remained in the solution for 10 min at room temperature and were then washed with PBS × 3. Next, the tissues were incubated with a primary antibody overnight at 4 °C. On the second day, after being washed with PBS for 5 min × 3, the sections were exposed to the secondary antibody at 37 °C for 1 h. The sections were subsequently stained with 3,3’-diaminobenzidine (DAB), counterstained with hematoxylin (SP9001, ZSZB-Bio, Beijing, China) for 2 min, and then rinsed in water for 20 min. All the tissue sections were photographed via a Leica DMI8 microscope. The immunohistochemistry (IHC)-stained sections were reviewed and scored independently by two experienced pathologists.

### Flow cytometry and cell sorting

Mouse tumors were dissociated into single-cell suspensions using a solution containing 2 mg/mL dispase (Solarbio, China), 1 mg/mL DNase IV (Solarbio, China) and 2 mg/mL collagenase (Solarbio, China). The cell suspension was then filtered through a 100 µm filter and washed with 1% BSA buffer. The cells were incubated with cell surface markers for 30–45 min at room temperature. Following incubation, the cells were fixed and permeabilized for intracellular staining according to the manufacturer’s protocol (BioLegend, USA). Single-cell suspensions from both tumors and splenocytes were stained according to a standard flow cytometry protocol and analyzed via a flow cytometer instrument (Sony, Japan). The antibodies used for this analysis are listed in the Results section. Flow cytometry data were analyzed via FlowJo™ Software v10.0 (Becton, Dickinson and Company, Ashland OR, USA).

### Statistical analyses

All the quantitative experiments were repeated for at least three independent biological replicates. The data are presented as the means ± SEMs or the means ± SDs. Statistical comparisons were performed via Student’s *t* test for comparisons between two groups or one-way ANOVA for multiple comparisons via GraphPad Prism 8.0 software (GraphPad Software, Inc., San Diego, CA, USA). In the figures, statistical significance is denoted as follows: *P < 0.05, **P < 0.01, ***P < 0.001, ****P < 0.0001, ns: not significantly different.

## Results

### YTHDF3 facilitates HPV carcinogenesis

**Fig. 1:**
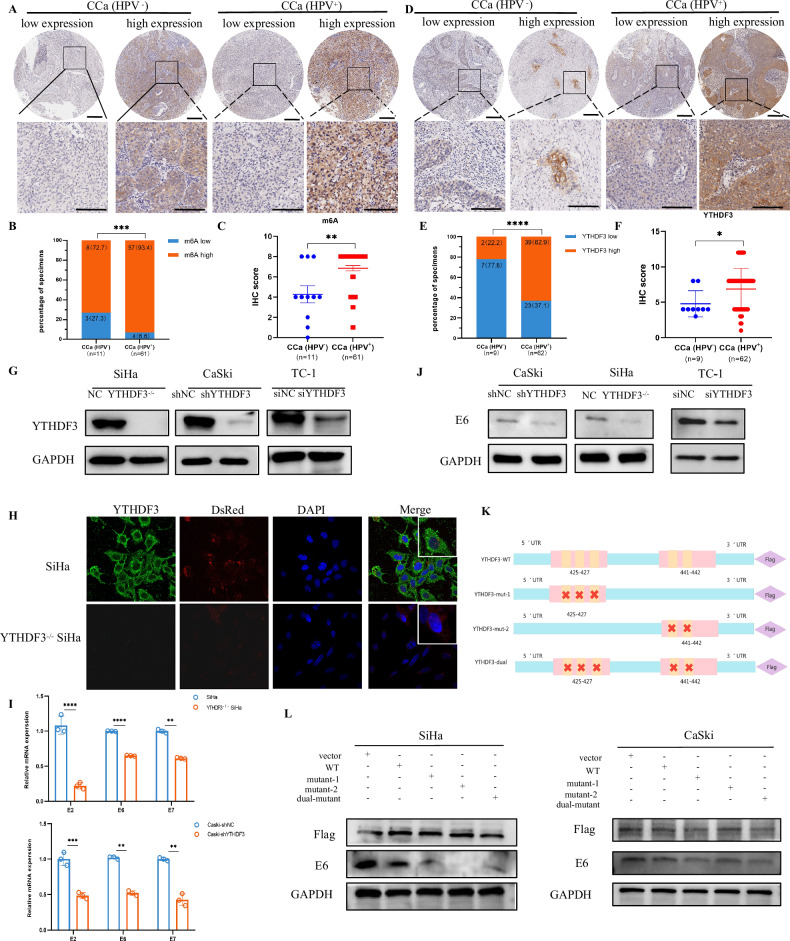
YTHDF3 facilitates HPV carcinogenesis. **A** Representative immunohistochemical (IHC) images displaying low and high expression of m^6^A in HPV^+^ CCa and HPV^-^ CCa patients in the TMA. Notably, the m^6^A modification level was lower in HPV^-^ CCa patients than in HPV^+^ CCa patients. The original magnification was 40× with a scale bar of 250 µm. The image below shows an outline of each image at a higher magnification view of the area, with a scale bar of 125 µm. **B** The proportion of patients with HPV^+^ CCa (*n* = 61) compared with HPV^-^ CCa (*n* = 11) revealed that HPV was associated with increased m^6^A modification in a clinical analysis of the TMA. Chi-square tests were used in this study. **C** The IHC score indicated that the HPV^+^ group had higher levels of m^6^A modification than did the HPV^-^ group. **D** Representative IHC images revealed low and high expression of YTHDF3 in HPV^+^ CCa and HPV^-^ CCa patients in the TMA. The original magnification was 40× with a scale bar of 250 µm. The image below shows an outline of each image at a higher magnification view of the area, with a scale bar of 125 µm. **E** The proportion of patients with HPV^+^ CCa (*n* = 62) compared with that with HPV^-^ CCa (*n* = 9) showed that HPV was associated with increased YTHDF3 expression in a clinical analysis of the TMA. Chi-square tests were used in this study. **F** The IHC score indicated that the HPV^+^ group had higher levels of m^6^A modification than did the HPV^-^ group. **G** Western blot analysis revealed the efficiency of YTHDF3 knockdown in SiHa, CaSki and TC-1 cells. **H** Immunofluorescence assays revealed the expression and location of YTHDF3 (green fluorescence) and HPV DsRed (red fluorescence) in SiHa cells and YTHDF3^-/-^ SiHa cells transfected with the HPV-DsRed plasmid. Original magnification 60×. **I** qPCR analysis demonstrated the RNA levels of the genes E1, E2, E6, and E7 following YTHDF3 silencing in SiHa and CaSki cells. The relative mRNA levels of these genes were normalized to that of GAPDH and were significantly downregulated. These results are expressed as the means ± SEMs of three independent experiments. **J** The protein expression levels of E6 in SiHa, CaSki and TC-1 cells were evaluated via western blotting. **K** The models show the generated FLAG-tagged mutant constructs, including YTHDF3-wild-type, YTHDF3-mut-1 (425–427), YTHDF3-mut-2 (441–442), and YTHDF3-dual (425–427, 441–442), with the vector as a control. **L** The protein levels of E6 in SiHa and CaSki cells transfected with wild-type or mutant Flag-tagged YTHDF3 were measured via western blotting. Absolute IHC scores were calculated as the intensity divided by the percentage of stained cells. The data are shown as the means ± SDs; *p < 0.05; **p < 0.01; *** p < 0.001; **** p < 0.0001; ns nonsignificant.

Persistent hr-HPV infection is a well-established driver of cervical carcinogenesis [10]. Our previous work demonstrated YTHDF3 upregulation in CCa facilitated lymph node metastasis [9]. While YTHDF3 was reported to suppress interferon signaling via FOXO3 translation [5], its role in anti-HPV infection remain undefined. To explore YTHDF3’s function in HPV carcinogenesis, we initially examined its expression in tissue microarray (TMA) samples obtained from both HPV-positive (HPV^+^) and HPV-negative (HPV^-^) CCa patients. Representative IHC images revealed low and high expression of m^6^A and YTHDF3 in HPV^+^ and HPV^-^ CCa patients. Notably, higher levels of m^6^A (Fig. 1A) and YTHDF3 expression (Fig. 1D) were observed in the HPV^+^ group than in the HPV^-^ group. Additionally, the positive expression rates of m^6^A (Fig. 1B) and YTHDF3 (Fig. 1E) in the HPV^+^ group were high (93.4%, 57/61 and 62.9%, 39/62, respectively) compared with those in the HPV^-^ group (72.7%, 8/11 and 22.2%, 2/9, respectively). The IHC scores for m^6^A (Fig. 1C) and YTHDF3 (Fig. 1F) were also elevated in the HPV^+^ group. Our analysis revealed notable increases in m^6^A modification levels and YTHDF3 expression in HPV^+^ patients compared with their HPV^-^ counterparts, establishing a positive correlation between m^6^A modification levels, YTHDF3 expression, and HPV infection. We then engineered CRISPR-Cas9-mediated YTHDF3-knockout (YTHDF3^−/−^) SiHa cells and validated the knockdown efficiency via western blot assays (Fig. 1G). Immunofluorescence imaging (Fig. 1H) showed reduced DsRed2-HPV nuclear localization in YTHDF3-deficient cells, suggesting impaired viral genome integration. The HPV genome comprises early regions and late regions. The early regions, including E2, E1, E6, and E7, are responsible for viral replication and transcription. Importantly, the integration of the HPV E7 and E6 oncogenes into the host genome is considered to be a key step in CCa development. These genes encode major malignant transforming proteins in oncogenic HPVs by binding the p53 or Rb genes. To further investigate the effect of YTHDF3 on HPV integration, we performed YTHDF3 knockdown or knockout experiments. Mechanistically, qRT-PCR analysis revealed downregulated E6/E7 transcripts in YTHDF3-knockout cells (Fig. 1I). And western blotting confirming selective E6 protein suppression (Fig. 1J), while E7 expression remained unchanged in both SiHa and CaSki cells(Supplementary Fig. 1), suggesting that YTHDF3 may promote HPV carcinogenesis by facilitating the integration of the HPV genome into the host genome, with a notable impact on E6 oncogene expression. To elucidate the role of the YTHDF3 RNA-binding domain in HPV genome integration, we generated FLAG-tagged mutant constructs, including YTHDF3-mut-1 (425–427), YTHDF3-mut-2 (441–442), and YTHDF3-dual (425--427, 441–442), along with the vector and YTHDF3-wild-type as controls. These constructs were then transfected into YTHDF3^+/+^ SiHa and CaSki cells (Fig. 1K). Notably, in CCa cells (SiHa/CaSki), while YTHDF3 mutant constructs (mut1 and mut2) exhibited reduced HPV E6 protein expression (Fig. 1L), the WT YTHDF3 group showed E6 levels comparable to the vector control. This observation is consistent with the functional role of YTHDF3’s RNA-binding domain in promoting E6 expression: the mutant constructs, lacking critical RNA-binding residues, fail to enhance E6 levels, whereas WT YTHDF3 overexpression does not further upregulate E6 beyond the baseline observed in the vector control (which likely reflects endogenous YTHDF3 activity in CCa cells). This observation is consistent with our previous findings showing that YTHDF3 knockout reduces E6 expression (Fig. 1J), indicating that YTHDF3’s contribution to E6 expression is context-dependent—required for basal E6 levels under conditions of low endogenous YTHDF3 expression (such as in knockout cells), but not sufficient to drive further upregulation when endogenous YTHDF3 is already present at high levels (as in WT-overexpressing CCa cells). To further validate this, we introduced the full-length HPV genome carrying the DsRed reporter gene (HPV-DsRed) into both YTHDF3-deficient cells and wild-type CCa cells. Notably, YTHDF3 deficiency significantly suppressed HPV infection efficiency and viral genome integration, accompanied by decreased E6 expression. These findings suggest that YTHDF3 facilitates a cellular environment conducive to HPV genome integration, and its deficiency disrupts this process, leading to reduced E6 expression and attenuated oncogenesis, which provides valuable insights into HPV-related carcinogenesis mechanisms.

### YTHDF3 negatively regulates type I ISGs in CCa

**Fig. 2:**
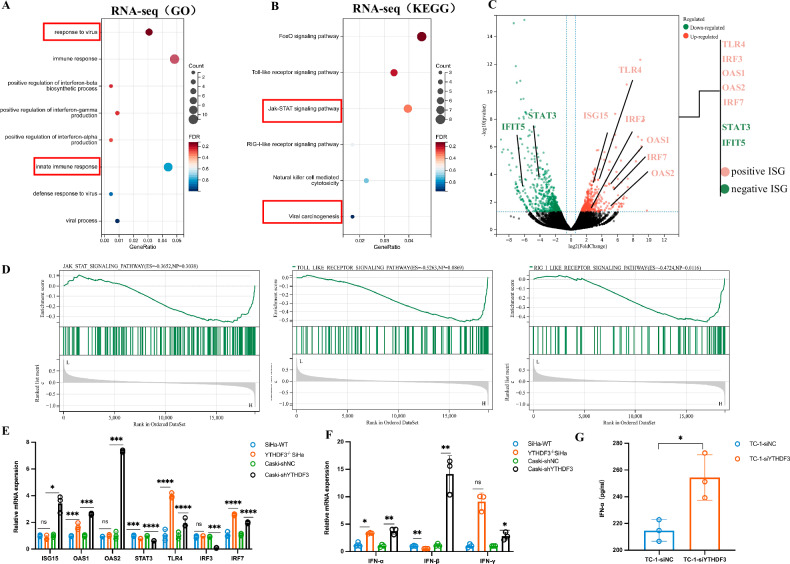
YTHDF3 negatively regulates type I ISGs in CCa. **A** GO analysis indicated that the DEGs were enriched in response to the virus, immune response, and positive regulation of IFN-I signaling pathways in the RNA-seq data. **B** KEGG pathway analysis of the RNA-seq data confirmed that the DEGs were enriched in the JAK-STAT signaling pathway. **C** Volcano plot showing differentially expressed genes, which included positive regulation ISGs (red color) and negative regulation ISGs (green color), upon knockout of YTHDF3 in SiHa cells. **D** Gene set enrichment analysis (GSEA) revealed that YTHDF3-associated genes were significantly enriched in the Toll-like signaling pathway, RIG-like signaling pathway, and JAK-STAT signaling pathway. **E** Real-time qPCR assays were used to compare the RNA levels of ISGs, including ISG15, OAS1, OAS2, STAT3, TLR4, IRF3, and IRF7, in the YTHDF3-knockout SiHa and CaSki cells with those in the control cells. The relative mRNA levels were normalized to that of GAPDH. The results are presented as the means ± SEMs of three independent assays. *p < 0.05, ***p < 0.001, ****p < 0.0001. **F** Real-time qPCR analysis was performed to measure the mRNA levels of type I interferon (IFN) signaling pathway components, including IFN-α, IFN-β, and IFN-γ, in SiHa cells with YTHDF3 knocked out via CRISPR-Cas9 (YTHDF3^−/−^ SiHa), negative control SiHa cells, shYTHDF3-expressing CaSki cells and shNC-expressing CaSki cells (control). The relative mRNA levels were normalized to the GAPDH mRNA levels. The results are expressed as the means ± SEMs of three independent experiments. *p < 0.05, **p < 0.01. **G** IFN-α production in supernatants harvested from siYTHDF3-TC-1 cells and negative control cells. The results are representative of at least two independent experiments.

Through comparative RNA sequencing (RNA-seq) analysis of wild-type versus YTHDF3-deficient SiHa cells, we identified distinct immunoregulatory signatures. Gene Ontology (GO) enrichment demonstrated that differentially expressed genes (DEGs) predominantly clustered within innate immune responses pathways, particularly interferon-alpha/beta/gamma-mediated signaling cascades (Fig. 2A). Kyoto Encyclopedia of Genes and Genomes (KEGG) analysis confirmed significant enrichment in both JAK-STAT and the RIG-I-like receptor signaling pathway (Fig. 2B). Volcano plot visualization of RNA-seq data highlighted polarized gene expression patterns following YTHDF3 ablation, with activation-linked ISGs (TLR4, IRF3, OAS1, OAS2, IRF7) showing marked upregulation and inhibitory ISGs (STAT3, IFIT5) being downregulated (Fig. 2C). Consistent with these findings, Gene set enrichment analysis (GSEA) reinforced the coordinated activation of JAK-STAT, RIG-I, and Toll-like receptor signaling pathways (Fig. 2D). Real-time qPCR analysis revealed that YTHDF3 deficiency significantly upregulated the key IFN-I response amplifier IRF7 in both SiHa and CaSki cells. While TLR3 and TLR4 exhibited consistent upregulation, cell type-dependent variations emerged: IRF3 was downregulated in CaSki cells, and no significant changes were observed for ISG15/OAS2 in SiHa cells (Fig. 2E). Importantly, STAT3 suppression persisted across all experimental systems, corroborating transcriptome-wide antiviral pathway activation (Fig. 2C). Mechanistic exploration demonstrated YTHDF3 knockdown potently induced IFN-α and IFN-β mRNA overexpression in cervical cancer cells (Fig. 2F). Functional validation through ELISA quantification of IFN-α secretome in TC-1 cells transfected with YTHDF3-targeting siRNA (siYTHDF3) versus non-targeting siRNA (siNC) revealed substantially elevated cytokine production (Fig. 2G). Collectively, these results establishes YTHDF3 as a potent suppressor of ISG networks in cervical carcinogenesis.

### YTHDF3 promotes STAT3 mRNA stability and translation efficiency in a manner dependent on METTL3-mediated m^6^A modification

**Fig. 3:**
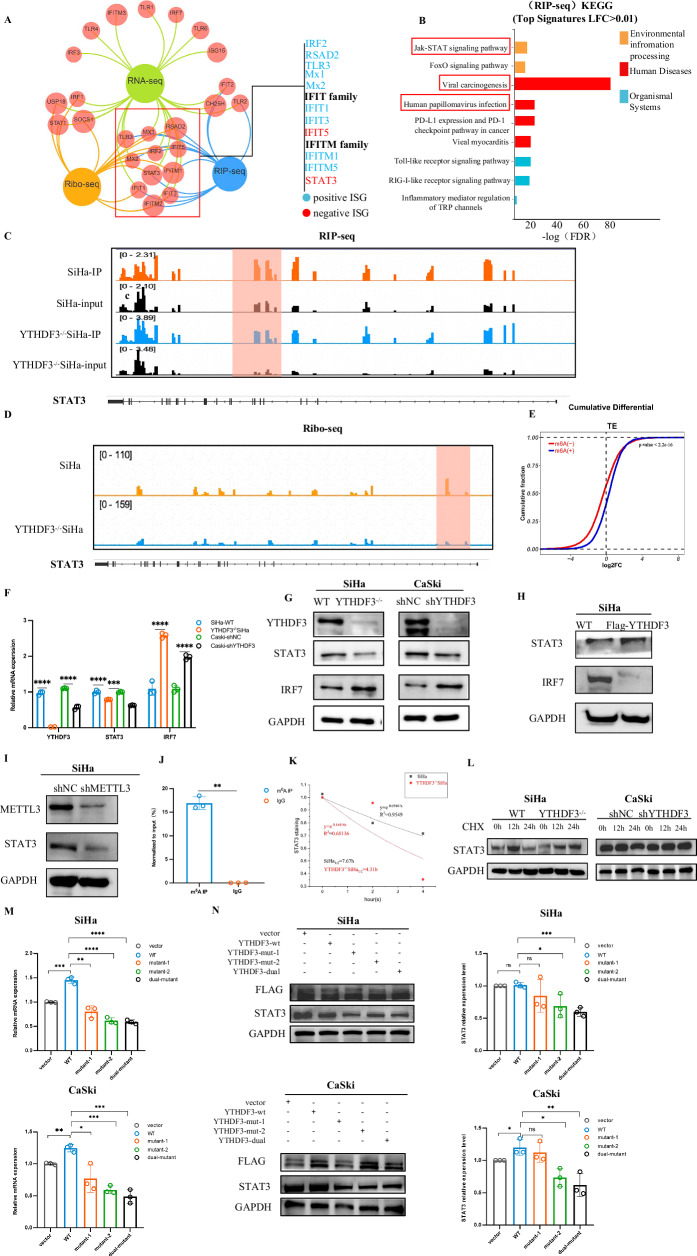
YTHDF3 promotes STAT3 mRNA stability and translation efficiency in a manner dependent on METTL3-mediated m^6^A modification. **A** Overlapping genes identified by Ribo-seq, RIP-seq, and RNA-seq were analyzed to identify YTHDF3 targets. **B** KEGG pathway analysis of the RIP-seq data revealed enriched pathways associated with the YTHDF3-binding genes. **C** Integrative Genomics Viewer (IGV) image showing the YTHDF3 binding site in the STAT3 mRNA identified by RIP-seq in SiHa and YTHDF3^−/−^ SiHa cells. **D** An IGV image of the Ribo-seq data confirms the peak enrichment of STAT3 mRNA, suggesting that YTHDF3 binding may enhance its translation. **E** Ribo-seq analysis revealed altered translation efficiency upon YTHDF3 knockout in SiHa cells. **F** qPCR analysis was used to compare the relative mRNA levels of STAT3 and IRF7 in the YTHDF3-knockdown SiHa and CaSki cells with those in their respective negative controls. The means and standard deviations from at least three independent experiments are shown. ***p < 0.001, ****p < 0.0001. **G** Western blot analysis revealed the protein levels of STAT3 and IRF7 in the YTHDF3-knockdown SiHa and CaSki cells compared with their respective negative controls. **H** Western blot analysis was used to assess STAT3 and IRF7 expression in the YTHDF3-deficient SiHa cells transfected with the YTHDF3 overexpression plasmid versus those transfected with the wild-type plasmid. **I** Western blot analysis was used to investigate whether STAT3 expression depends on METTL3-mediated m^6^A modification by comparing STAT3 protein levels in shMETTL3 SiHa and shNC SiHa cells (sh denotes short hairpin RNA-mediated knockdown). **J** MeRIP-qPCR results showing the m^6^A enrichment of STAT3 mRNA. The means and standard deviations from at least two independent experiments are displayed. **p < 0.01. **K** qPCR assays were performed to determine the effect of YTHDF3 on STAT3 mRNA stability after SiHa cells and YTHDF3^−/−^ SiHa cells were treated with 4 µg/mL actinomycin D, and the cells were harvested at 0, 2, and 4 h. The decay rate for each transcript was calculated via the exponential functions of Half-Life. The means and standard deviations from at least three independent experiments are shown. **L** Western blot analysis showing the effect of YTHDF3 on the protein stability of STAT3 after SiHa and YTHDF3^−/−^ SiHa cells and shNC CaSki and shYTHDF3 CaSki cells were challenged with 200 µg/mL CHX and harvested at 0, 12, and 24 h. **M** qPCR analysis comparing STAT3 mRNA levels in SiHa and CaSki cells after transfection with vector, wild-type or mutant Flag-tagged YTHDF3 plasmids. The means and deviations from at least two independent experiments are displayed. The data are shown as the means ± SDs; *p <0.05;**p < 0.01; ***p < 0.001; **** p < 0.0001. **N** Western blot analysis of STAT3 protein levels in SiHa and CaSki cells transfected with vector, wild-type or mutant Flag-tagged YTHDF3 plasmids. STAT3 expression was normalized with GAPDH. The mean ± SD from three experiments was plotted. ns no significant difference, *p < 0.05, **p < 0.01, ***p < 0.001. GAPDH was used as loading control.

To further characterize the key downstream targets of YTHDF3 and dissect its mechanism in regulating the IFN-I response, we employed a multi-omics approach integrating RNA immunoprecipitation sequencing (RIP-seq), RNA-seq, and ribosome sequencing (Ribo-seq) techniques in both SiHa and YTHDF3^−/^^−^ SiHa cells. This integrative analysis identified 11 commonly enriched genes, including STAT3, a critical member of the JAK-STAT pathway that plays a crucial role in viral processes and oncogenesis [11] (Fig. 3A). KEGG pathway analysis of our RIP-seq data revealed significant overrepresentation of YTHDF3-bound transcripts in JAK-STAT signaling and viral carcinogenesis pathways (Fig. 3B), implicating YTHDF3-mediated gene regulation in adaptive immunity and interferon signaling, particularly through JAK-STAT and RIG-like receptor pathways. Following this extensive analysis, STAT3 was identified as the gene of interest for subsequent investigation because of its pivotal role, as suggested by our preliminary findings. RIP-seq confirmed that the STAT3 gene was bound by the YTHDF3 protein, with over twofold greater enrichment than the size-matched control (Fig. 3C). Ribo-seq data visualized by IGV software demonstrated impaired STAT3 translational efficiency in the YTHDF3^−/−^ SiHa cells (Fig. 3D), with global Ribo-seq analysis indicating m^6^A modification influences overall translational output in SiHa cells (Fig. 3E). A recent study in large B-cell lymphoma showed STAT3 negatively regulates the IFN-I signaling pathway by suppressing IRF7 expression to inhibit cell death [12], promoting us to hypothesize a YTHDF3-STAT3-IRF7 regulatory axis. Indeed, YTHDF3 deficiency significantly reduced STAT3 protein/mRNA levels while increasing IRF7 expression compared to controls (Fig. 3F, G), effects reversed by stable reexpression of FLAG-YTHDF3 in YTHDF3^−/−^ cells (Fig. 3H).

To assess the role of METTL3 -the canonical m^6^A methyltransferase- in STAT3 regulation, we generated stable METTL3-knockdown SiHa cells using shMETTL3 lentivirus. Western blot analysis revealed reduced STAT3 protein levels upon METTL3 silencing, indicating STAT3 expression is dependent on METTL3-mediated m^6^A modification (Fig. 3I). MeRIP-qPCR confirmed specific m^6^A enrichment in STAT3 mRNA, with significantly higher modification levels in anti-m^6^A IP samples compared to IgG controls (Fig. 3J). Actinomycin D chase assays showed accelerated STAT3 mRNA decay in YTHDF3^−/−^ cells, demonstrating YTHDF3 maintains STAT3 mRNA stability (Fig. 3K). Cycloheximide (CHX) chase experiments, however, revealed no difference in STAT3 protein turnover between YTHDF3-knockdown and control cells, ruling out effects on proteolytic degradation (Fig. 3L).

Functional domain mapping using plasmids expressing wild-type YTHDF3 (YTHDF3-wt), YTH domain-deficient mutants (YTHDF3-mut1, YTHDF3-mut2, YTHDF3-dual), or empty vector in SiHa and CaSki cells revealed distinct regulatory patterns. While mutant constructs (mut1, mut2, dual) exhibited reduced STAT3 protein levels compared to YTHDF3-wt and vector controls (Fig.3M, N), overexpression of WT YTHDF3 in CaSki cells (which exhibit lower endogenous YTHDF3 expression) led to a 1.34-fold increase in STAT3 protein compared to vector controls. In contrast, STAT3 levels in SiHa cells (with high endogenous YTHDF3) remained comparable to vector controls, suggesting a context-dependent regulatory mechanism. This differential response aligns with our mechanistic findings: YTHDF3 promotes STAT3 expression through m6A-dependent mRNA stabilization and translation, a process requiring an intact YTH domain. In cells with saturated endogenous YTHDF3 activity (e.g., SiHa), exogenous WT YTHDF3 fails to further enhance STAT3 due to a ceiling effect. Conversely, in YTHDF3-deficient or low-expressing cells (e.g., CaSki), WT YTHDF3 reintroduces m6A-binding capacity, restoring STAT3 expression. Collectively, these findings establish that YTHDF3 promotes STAT3 expression through METTL3-dependent m^6^A modification, enhancing mRNA stability and subsequent translational efficiency - a mechanism that culminates in suppression of the IRF7/IFN-I signaling cascade.

### STAT3 is pivotal in the YTHDF3-mediated negative regulation of Type I IFNs in HPV carcinogenesis

**Fig. 4:**
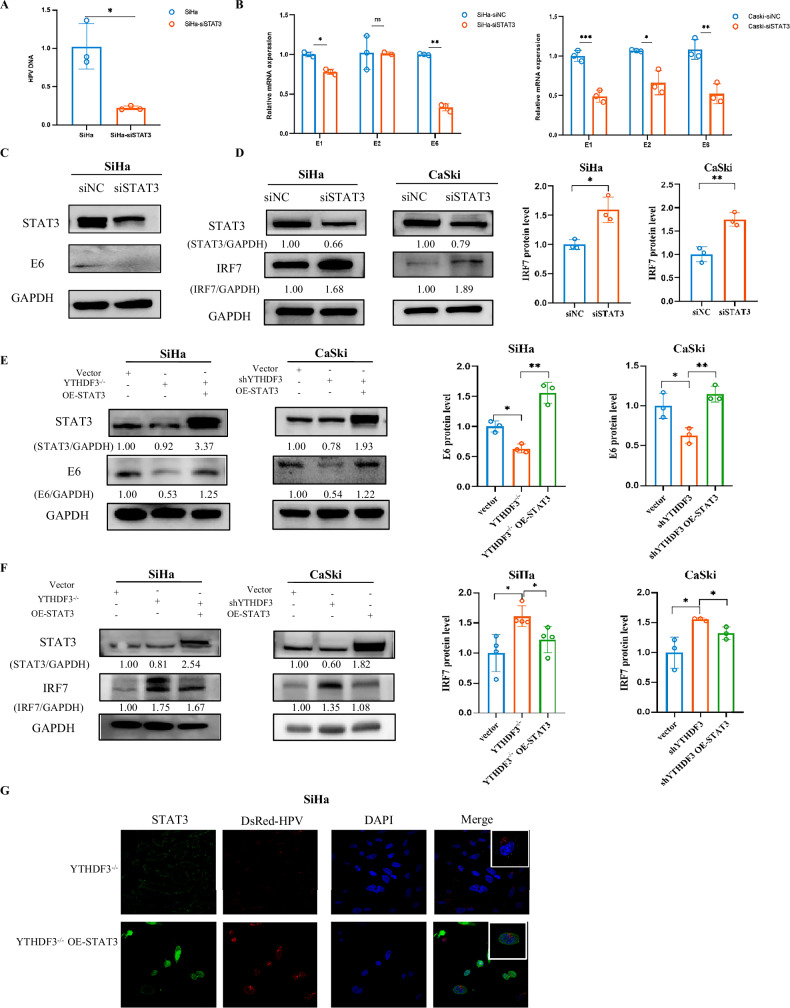
STAT3 is pivotal in the YTHDF3-mediated negative regulation of HPV carcinogenesis by type I IFNs. **A** qPCR analysis was used to measure HPV DNA levels in SiHa cells transfected with either siSTAT3 (STAT3 siRNA) or siNC (negative control siRNA). The data are presented as the means ± SEMs of two independent experiments. *p < 0.05. **B** qPCR analysis of the mRNA levels of the HPV E1, E2, and E6 genes in siSTAT3-transfected cervical cancer cells compared with those in negative control cells. The data are presented as the means ± SEMs of two independent experiments. *p < 0.05, **p < 0.01. **C** Western blot analysis showing E6 protein expression in SiHa cells transfected with either a control vector or siSTAT3 for 48 h. **D** Western blot analysis of STAT3 and IRF7 protein levels in SiHa and CaSki cells transfected with either a control vector or siSTAT3 for 48 h. STAT3 and IRF7 expression was normalized with GAPDH. Bar graphs exhibited normalized IRF7 expressions from 3 independent experiments. Data were presented as mean ± SD from 3 independent experiments. *P* values were calculated by Student’s *t* test, indicating *p < 0.05, **p < 0.01. GAPDH was used as loading control. **E** Western blot analysis was used to assess E6 protein expression in CCa cells following transfection with a control vector, a YTHDF3 knockdown (siRNA), a STAT3 overexpression plasmid, or cotransfection with a YTHDF3 knockdown and STAT3 overexpression plasmid. The cells were incubated for 48 h after transfection. STAT3 and E6 expression was normalized with GAPDH. Bar graphs exhibited normalized E6 expressions from 3 independent experiments. Data were presented as mean ± SD from 3 independent experiments. *P* values were calculated by Student’s t-test, indicating *p < 0.05, **p < 0.01. GAPDH was used as loading control. **F** Western blot analysis of STAT3 and IRF7 protein levels in YTHDF3 knockout (YTHDF3^−/−^) CCa cells with STAT3 overexpression (OE-STAT3) compared with control YTHDF3^−/−^ cells. STAT3 and IRF7 expression was normalized with GAPDH. Bar graphs exhibited normalized IRF7 expressions from 3 or 4 independent experiments. Data were presented as mean ± SD from 3 or 4 independent experiments. P values were calculated by Student’s t-test, indicating *p < 0.05. GAPDH was used as loading control. **G** Immunofluorescence assays were used to visualize the location and expression of YTHDF3 (green fluorescence) and HPV DsRed (red fluorescence) in YTHDF3^−/−^ SiHa cells and YTHDF3^−/−^ SiHa cells overexpressing STAT3 (YTHDF3^−/−^ OE-STAT3). Original magnification, 60×; scale bar = 100 µm.

Emerging evidence establishes STAT3 as a key modulator of antiviral innate immunity through ISG regulation [13]. Building on this paradigm, we systematically interrogated STAT3’s pathogenic involvement in HPV-driven carcinogenesis via ISG networks. Pharmacogenetic silencing of STAT3 using sequence-specific siRNA (siSTAT3) produced dual therapeutic effects: significantly reduced HPV infection rates and concomitant suppression of E6 oncogene expression at both transcriptional and translational levels (Fig. 4A–C), pinpointing STAT3’s direct pro-carcinogenic role in HPV genomic integration. Mechanistic dissection through immunoblotting uncovered STAT3’s transcriptional regulation of IRF7, the master coordinator of antiviral responses (Fig. 4D). Rescue experiments in YTHDF3-deficient cells confirmed the specificity of STAT3’s role: STAT3 overexpression restored STAT3 protein levels (Fig. 4E) and reversed YTHDF3-mediated suppression of E6 (Fig. 4E), while concomitantly inhibiting IRF7 activation (Fig. 4F). This reciprocal relationship between STAT3 and IRF7 aligns with our proposed model, where STAT3 acts as a downstream antagonist of the IRF7/IFN-I axis. Strikingly, HPV-DsRed tracking assays revealed STAT3 reconstitution rescued nuclear translocation efficiency of HPV particles in YTHDF3^−/−^ SiHa cells (Fig. 4G), quantitatively restoring infectivity parameters to near-wild-type levels. Taken together, our study demonstrated that YTHDF3-mediated STAT3 acts as a negative regulator of the IRF7/IFN-I signaling pathway, playing a pivotal role in counteracting HPV-induced carcinogenesis.

### YTHDF3/m^6^A/STAT3 suppress antitumor immunity and create an ITME

**Fig. 5:**
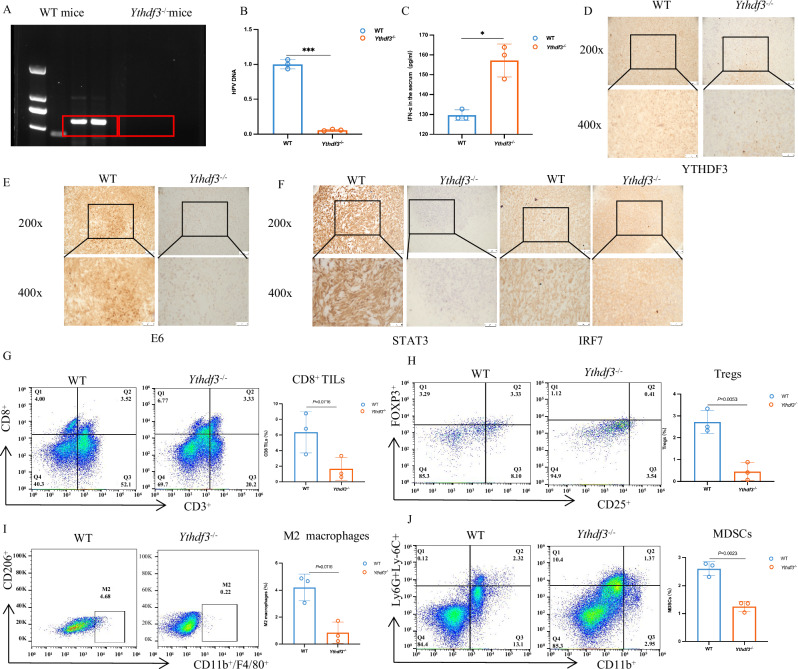
YTHDF3/m^6^A/STAT3 suppresses antitumor immunity and creates an ITME. **A** PCR analysis validated the knockout efficiency of the *Ythdf3* gene in *Ythdf3*^−/−^ mice (*n* = 2). **B** Viral titers in tumor tissues collected on day 10 postinfection were measured via qPCR in *Ythdf3*^*+/+*^ and *Ythdf3*^−/−^ mice, and HPV DNA levels were reduced in the latter group (*n* = 3). **C** The production of IFN-α in the serum of wild-type (WT) mice and *Ythdf3*^−/−^mice was measured (*n* = 3). **D** IHC staining was used to visualize YTHDF3 protein expression in the tumor tissues of wild-type (WT) mice and *Ythdf3*^−^^/^^−^ mice (*n* = 3). **E** IHC analysis of E6 protein expression in tumor tissues from wild-type (WT) mice and *Ythdf3*^−^^/^^−^ mice (*n* = 3). **F** IHC analysis of STAT3 and IRF7 protein expression in the tumor tissues of *Ythdf3*^−^^/^^−^ mice and control mice. **G** The percentage of CD8^+^ T cells in the tumor tissues of *Ythdf3*^−/−^ mice and wild-type mice was determined (*n* = 3). **H** The percentages of Tregs in *Ythdf3*^−/−^ and wild-type mice were evaluated (*n* = 3). **I** The frequency of M2 macrophages was detected in the tumor tissues of *Ythdf3*^−/−^mice and wild-type mice (*n* = 3). **J** The percentage of MDSCs in the tumor tissues of *Ythdf3*^−/−^ mice and wild-type mice (*n* = 3).

To delineate the immunoregulatory hierarchy of the YTHDF3/m6A/STAT3 axis in tumor-immune crosstalk, we engineered *Ythdf3* knockout (*Ythdf3*^−/−^) mice through CRISPR/Cas9-mediated editing and established TC-1 xenograft models. PCR assays validated the *Ythdf3* knockout efficiency, confirming the successful generation of *Ythdf3*^−/−^ mice (Fig. 5A). Importantly, *Ythdf3* ablation substantially attenuated HPV viral burden (Fig. 5B), impairing viral persistence within the tumor niche. Systemic immune profiling revealed heightened serum IFN-α levels in *Ythdf3*^−/−^ mice (Fig. 5C), corroborating enhanced IFN-I activation. Similarly, immunohistochemical analysis revealed lower protein levels of YTHDF3 and E6 in tumors from *Ythdf3*^−/−^ mice than in those from their wild-type counterparts. These tumors also presented decreased STAT3 and increased IRF7 protein expression (Fig. 5D–F). These findings provide compelling evidence that YTHDF3 deficiency significantly suppresses antitumor immunity while simultaneously activating IFN-I signaling pathways in vivo.

It is well established that innate immunity plays a key role in initiating tumor immunity [3]. A recent study identified STAT3 as a critical mediator in various immune cell populations, including natural killer (NK) cells, regulatory T cells (Tregs), and myeloid cells [14]. Despite CD8^+^ T cells’ canonical role in antiviral surveillance [15], *Ythdf3*^*-/-*^ mice maintained intriguingly preserved CD8^+^ T cell infiltration (Fig. 5G). On the basis of these findings, we hypothesized that YTHDF3 might regulate the development of the ITME by influencing the accumulation of tumor-associated macrophages (TAMs), myeloid-derived suppressor cells (MDSCs), and Tregs. Therefore, we conducted a comparative analysis to assess the proportions of MDSCs, M2 macrophages, and Tregs in *Ythdf3*^−/−^ mice compared with those in wild-type controls. Flow cytometry data clearly revealed a reduction in Treg cells in *Ythdf3*^−/−^ mice (Fig. 5H). We next examined the infiltration levels of macrophages in *Ythdf3*^−/−^ mice and wild-type mice. Importantly, YTHDF3 deficiency resulted in a significant decrease in M2 macrophages (CD11b^+^/F4/80^+^/CD206^+^) and promoted the shift of M2 macrophages toward the M1 phenotype (Fig. 5I). Finally, the number of MDSCs (CD11b^+^/LY-6G/LY-6C^+^) isolated from the *Ythdf3*^−/−^ group was markedly lower than that isolated from the control group (Fig. 5J). In summary, our findings demonstrated that YTHDF3 silencing not only enhances the antiviral response in vivo by activating IFN-I signaling but also strengthens antitumor immunity by suppressing the formation of an ITME characterized by Treg infiltration, M2 macrophage recruitment, and MDSC accumulation.

## Discussion

CCa is a leading cause of mortality among women, underscoring the urgent need for improved therapeutic strategies [16]. The dysregulation of m^6^A RNA modification has emerged as a key contributor to tumor progression and clinical outcomes. Targeting m^6^A regulatory components, particularly the reader protein YTHDF3, thus presents a viable strategy for anticancer intervention.

Viral genome integration constitutes the molecular linchpin of HPV-driven oncogenesis [7, 17, 18]. Diverging from prior reports on YTHDF3’s role in *B. mori* nuclear polyhedrosis virus (BmNPV) infection [19], our study highlights its crucial involvement in HPV^+^ CCa. Clinical-pathological analyses revealed YTHDF3 overexpression signatures in HPV^+^ CCa cohorts. Functional genomics demonstrated YTHDF3’s proviral activity through E6 oncoprotein stabilization, recapitulated in *Ythdf3*^*−/−*^ xenografts showing sustained HPV suppression. Single-particle tracking using HPV-DsRed constructs conclusively demonstrated that YTHDF3 ablation impedes viral nuclear translocation, thereby compromising integration competence.

IFN-I, principally IFN-α and IFN-β, exhibit a diverse range of antiviral, antiproliferative, antiangiogenic and immunostimulatory properties, mediating antitumor effects through the upregulation of ISGs [11, 20, 21]. YTHDF3 deficiency promotes basal ISG expression by inhibiting FOXO3 translation and regulating antiviral immunity [5]. Consistent with these observations, RNA-seq analysis of SiHa and YTHDF3^−/−^ SiHa cells revealed that YTHDF3 is involved in the IFN-I signaling pathway. Notably, YTHDF3 ablation not only amplified ISG transcription but also potentiated IFN-α secretion.

Integrative multi-omics analyses (RIP-seq, RNA-seq, and Ribo-seq) identified the JAK-STAT pathway as a key YTHDF3-regulated signaling cascade. Specifically, YTHDF3 was found to primarily target STAT3, a transcription factor that is frequently activated in human cancers and is involved in viral processes [14, 22]. Several studies have shown that STAT3 inhibits IFN-I-mediated antiviral responses in immune cells [23, 24]. Our mechanistic studies revealed that YTHDF3 enhances STAT3 mRNA stability, thereby attenuating IFNα production in malignant cells. Importantly, IFN-α exhibits potent antiviral and immunostimulatory properties, along with its antitumor-immune effects [25]. Moreover, it plays a crucial role in the positive feedback loop by amplifying the production of both IFN-α and IFN-β through IRF7, which is considered a pivotal ISG [12, 26]. Phosphorylated IRF7 triggers the production and secretion of IFN-I, while upregulated IFN-I activates the JAK1-STAT1/2 signaling pathway, subsequently activating ISG production [27]. In our previous study, we observed enhanced HPV integration following STAT3 rescue in YTHDF3-depleted cells, confirming YTHDF3’s immunomodulatory role through the STAT3/IRF7/IFN-I axis.

Accumulating evidence positions STAT3 as a master oncogenic regulator across malignancies through its suppression of IFN-I signaling [28]. Importantly, aberrantly activated STAT3 can mediate communication between cancer cells and their immunological microenvironment, leading to tumor-induced immunosuppression. Type I IFNs exert pleiotropic antitumor effects, including immunological [1], vascular, and direct antitumor effects [29, 30]. The immunological effects include contributions to dendritic cell maturation and antigen presentation, activation of memory CD8^+^ T cells, and inactivation of regulatory T cells [31–33]. Immunosuppressive cells such as Tregs, M2 macrophages and MDSCs play pivotal roles within the tumor microenvironment (TME) [34, 35]. Elucidating the epigenetic mechanism in both tumor and immune cells may offer promising therapeutic opportunities for combination therapies in CCa. Our experimental models demonstrated substantial depletion of these immunosuppressive elements in *Ythdf3*^−/−^ TMEs, revealing that YTHDF3 deficiency reprograms the immunotolerant tumor microenvironment. Collectively, these findings establish the YTHDF3/m^6^A/STAT3 axis as a multimodal regulator of antitumor immunity and TME composition in CCa.

## Conclusion

In summary, YTHDF3 promotes HPV-induced cervical carcinogenesis by stabilizing STAT3 mRNA via m^6^A binding. This, in turn, negatively regulates type I ISG expression and IFN-α production, facilitating the formation of an ITME in CCa (graphical abstract). Importantly, our findings highlight that YTHDF3 deficiency can significantly suppress the HPV carcinogenic process and potentiate the antitumor immune response. This study suggests novel therapeutic avenues for treating CCa patients by targeting the YTHDF3/STAT3/IFN-I signaling pathway.

## Supplementary information

Supplmentary Table 1 & 2

Supplemental Figure1

Original blots

## Data Availability

The datasets generated and/or analyzed during the current study are available from [GSE210342] and [GSE216813]. Full length western blots are showed in supplementary information files.

## References

[1] Yu R, Zhu B, Chen D. Type I interferon-mediated tumor immunity and its role in immunotherapy. Cell Mol Life Sci. 2022;79:191. 35292881 10.1007/s00018-022-04219-z PMC8924142

[2] Holicek P, Guilbaud E, Klapp V, Truxova I, Spisek R, Galluzzi L, et al. Type I interferon and cancer. Immunol Rev. 2024;321:115–27. 37667466 10.1111/imr.13272

[3] Feng M, Jiang W, Kim BYS, Zhang CC, Fu YX, Weissman IL. Phagocytosis checkpoints as new targets for cancer immunotherapy. Nat Rev Cancer. 2019;19:568–86. 31462760 10.1038/s41568-019-0183-z PMC7002027

[4] Li X, Ma S, Deng Y, Yi P, Yu J. Targeting the RNA m6A modification for cancer immunotherapy. Mol Cancer. 2022;21:76. 35296338 10.1186/s12943-022-01558-0 PMC8924732

[5] Zhang Y, Wang X, Zhang X, Wang J, Ma Y, Zhang L, et al. RNA-binding protein YTHDF3 suppresses interferon-dependent antiviral responses by promoting FOXO3 translation. Proc Natl Acad Sci USA. 2019;116:976–81. 30591559 10.1073/pnas.1812536116 PMC6338863

[6] Shi H, Wang X, Lu Z, Zhao BS, Ma H, Hsu PJ, et al. YTHDF3 facilitates translation and decay of N6-methyladenosine-modified RNA. Cell Res. 2017;27:315–28. 28106072 10.1038/cr.2017.15 PMC5339834

[7] Zur Hausen H. Papillomaviruses in the causation of human cancers - a brief historical account. Virology. 2009;384:260–5. 19135222 10.1016/j.virol.2008.11.046

[8] Mittal S, Banks L. Molecular mechanisms underlying human papillomavirus E6 and E7 oncoprotein-induced cell transformation. Mutat Res Rev Mutat Res. 2017;772:23–35. 28528687 10.1016/j.mrrev.2016.08.001

[9] Zhong S, Guo Q, Chen X, Luo X, Long Y, Chong T, et al. The inhibition of YTHDF3/m6A/LRP6 reprograms fatty acid metabolism and suppresses lymph node metastasis in cervical cancer. Int J Biol Sci. 2024;20:916–36. 38250152 10.7150/ijbs.87203 PMC10797697

[10] Jacot-Guillarmod M, Balaya V, Mathis J, Hübner M, Grass F, Cavassini M, et al. Women with cervical high-risk human papillomavirus: be aware of your anus! The ANGY cross-sectional clinical study. Cancers. 2022;14:5096. 36291879 10.3390/cancers14205096 PMC9600245

[11] El-Tanani M, Al Khatib AO, Aladwan SM, Abuelhana A, McCarron PA, Tambuwala MM. Importance of STAT3 signalling in cancer, metastasis and therapeutic interventions. Cell Signal. 2022;92:110275. 35122990 10.1016/j.cellsig.2022.110275

[12] Lu L, Zhu F, Zhang M, Li Y, Drennan AC, Kimpara S, et al. Gene regulation and suppression of type I interferon signaling by STAT3 in diffuse large B cell lymphoma. Proc Natl Acad Sci USA. 2018;115:E498–E505. 29295936 10.1073/pnas.1715118115 PMC5776985

[13] Mahony R, Gargan S, Roberts KL, Bourke N, Keating SE, Bowie AG, et al. A novel anti-viral role for STAT3 in IFN-α signalling responses. Cell Mol Life Sci. 2017;74:1755–64. 27988795 10.1007/s00018-016-2435-3 PMC11107673

[14] Piper M, Van Court B, Mueller A, Watanabe S, Bickett T, Bhatia S, et al. Targeting Treg-expressed STAT3 enhances NK-mediated surveillance of metastasis and improves therapeutic response in pancreatic adenocarcinoma. Clin Cancer Res. 2022;28:1013–26. 34862244 10.1158/1078-0432.CCR-21-2767 PMC8898296

[15] Klebanoff CA, Gattinoni L, Restifo NP. CD8+ T-cell memory in tumor immunology and immunotherapy. Immunol Rev. 2006;211:214–24. 16824130 10.1111/j.0105-2896.2006.00391.x PMC1501075

[16] Small W Jr, Bacon MA, Bajaj A, Chuang LT, Fisher BJ, et al. Cervical cancer: a global health crisis. Cancer. 2017;123:2404–12. 28464289 10.1002/cncr.30667

[17] Hu C, Liu T, Han C, Xuan Y, Jiang D, Sun Y, et al. HPV E6/E7 promotes aerobic glycolysis in cervical cancer by regulating IGF2BP2 to stabilize m6A-MYC expression. Int J Biol Sci. 2022;18:507–21. 35002506 10.7150/ijbs.67770 PMC8741847

[18] Crosbie EJ, Einstein MH, Franceschi S, Kitchener HC. Human papillomavirus and cervical cancer. Lancet. 2013;382:889–99. 23618600 10.1016/S0140-6736(13)60022-7

[19] Zhang X, Zhang Y, Dai K, Liang Z, Zhu M, Pan J, et al. N6-Methyladenosine level in silkworm midgut/ovary cell line is associated With *Bombyx mori* nucleopolyhedrovirus Infection. Front Microbiol. 2020;10:2988. 31998272 10.3389/fmicb.2019.02988 PMC6965365

[20] Zhou L, Zhang Y, Wang Y, Zhang M, Sun W, Dai T, et al. A dual role of type I interferons in antitumor immunity. Adv Biosyst. 2020;4:e1900237. 33245214 10.1002/adbi.201900237

[21] Schoggins JW, Wilson SJ, Panis M, Murphy MY, Jones CT, Bieniasz P, et al. A diverse range of gene products are effectors of the type I interferon antiviral response. Nature. 2011;472:481–5. 21478870 10.1038/nature09907 PMC3409588

[22] Johnson DE, O’Keefe RA, Grandis JR. Targeting the IL-6/JAK/STAT3 signaling axis in cancer. Nat Rev Clin Oncol. 2018;15:234–48. 29405201 10.1038/nrclinonc.2018.8 PMC5858971

[23] Sun L, Zhang X, Song Q, Liu L, Forbes E, Tian W, et al. IGFBP2 promotes tumor progression by inducing alternative polarization of macrophages in pancreatic ductal adenocarcinoma through the STAT3 pathway. Cancer Lett. 2021;500:132–46. 33309859 10.1016/j.canlet.2020.12.008 PMC7923838

[24] McFadden MJ, Sacco MT, Murphy KA, Park M, Gokhale NS, Somfleth KY, et al. FTO suppresses STAT3 activation and modulates proinflammatory interferon-stimulated gene expression. J Mol Biol. 2022;434:167247. 34537236 10.1016/j.jmb.2021.167247 PMC8924017

[25] Yang C, Zhao X, Sun D, Yang L, Chong C, Pan Y, et al. Interferon alpha (IFNα)-induced TRIM22 interrupts HCV replication by ubiquitinating NS5A. Cell Mol Immunol. 2016;13:94–102. 25683609 10.1038/cmi.2014.131 PMC4711679

[26] Ma F, Li B, Yu Y, Iyer SS, Sun M, Cheng G. Positive feedback regulation of type I interferon by the interferon-stimulated gene STING. EMBO Rep. 2015;16:202–12. 25572843 10.15252/embr.201439366 PMC4328747

[27] Kitagawa Y, Sakai M, Funayama M, Itoh M, Gotoh B. Human Metapneumovirus M2-2 protein acts as a negative regulator of alpha interferon production by plasmacytoid dendritic cells. J Virol. 2017;91:e00579-17. 28768858 10.1128/JVI.00579-17 PMC5625510

[28] Wang T, Niu G, Kortylewski M, Burdelya L, Shain K, Zhang S, et al. Regulation of the innate and adaptive immune responses by Stat-3 signaling in tumor cells. Nat Med. 2004;10:48–54. 14702634 10.1038/nm976

[29] Zitvogel L, Galluzzi L, Kepp O, Smyth MJ, Kroemer G. Type I interferons in anticancer immunity. Nat Rev Immunol. 2015;15:405–14. 26027717 10.1038/nri3845

[30] Enomoto H, Tao L, Eguchi R, Sato A, Honda M, Kaneko S, et al. The in vivo antitumor effects of type I-interferon against hepatocellular carcinoma: the suppression of tumor cell growth and angiogenesis. Sci Rep. 2017;7:12189. 28939881 10.1038/s41598-017-12414-3 PMC5610170

[31] Nefedova Y, Huang M, Kusmartsev S, Bhattacharya R, Cheng P, Salup R, et al. Hyperactivation of STAT3 is involved in abnormal differentiation of dendritic cells in cancer. J Immunol. 2004;172:464–74. 14688356 10.4049/jimmunol.172.1.464

[32] Nefedova Y, Nagaraj S, Rosenbauer A, Muro-Cacho C, Sebti SM, Gabrilovich DI. Regulation of dendritic cell differentiation and antitumor immune response in cancer by pharmacologic-selective inhibition of the janus-activated kinase 2/signal transducers and activators of transcription 3 pathway. Cancer Res. 2005;65:9525–35. 16230418 10.1158/0008-5472.CAN-05-0529 PMC1351362

[33] Brayer J, Cheng F, Wang H, Horna P, Vicente-Suarez I, Pinilla-Ibarz J, et al. Enhanced CD8 T cell cross-presentation by macrophages with targeted disruption of STAT3. Immunol Lett. 2010;131:126–30. 20346983 10.1016/j.imlet.2010.03.004 PMC2906450

[34] Oliver AJ, Davey AS, Keam SP, Mardiana S, Chan JD, von Scheidt B, et al. Tissue-specific tumor microenvironments influence responses to immunotherapies. Clin Transl Immunol. 2019;8:e1094. 10.1002/cti2.1094 PMC6869967 31768254

[35] Nishikawa H, Koyama S. Mechanisms of regulatory T cell infiltration in tumors: implications for innovative immune precision therapies. J Immunother Cancer. 2021;9:e002591. 34330764 10.1136/jitc-2021-002591 PMC8327843

