# Supplementary material for: YTHDF3 suppresses interferon-stimulated gene (ISG)-dependent antitumor immunity and promotes HPV carcinogenesis in cervical cancer
Source: Cell Death Dis. 2025 Dec 26;17(1):60. doi: 10.1038/s41419-025-08188-6 (PMC12827990; doi:10.1038/s41419-025-08188-6)
Supplement: Supplementary file 1 — Supplmentary Table 1 & 2 [file 41419_2025_8188_MOESM1_ESM.docx]

**Table S1. Antibodies used in the experiments.**

| **Antibody** | **Source** | **No. of Catalogue** | **Dilution** | | | |
| --- | --- | --- | --- | --- | --- | --- |
|  |  |  | **WB** | **IHC** | **IF** | **FCM** |
| N^6^-Methyladenoe | Abcam, USA | 220161 | 1:1000 | 1:100 |  |  |
| YTHDF3 | Abcam, USA | 190886 | 1:1000 | 1:500 | 1:200 |  |
| E6 | Bioss | 2560T | 1:500 | 1:1000 |  |  |
| L1 | Abcam, USA | A13324 | 1:1000 | 1:800 |  |  |
| STAT3 | Protein tech，China | 5741 | 1:1000 | 1:1000 |  |  |
| IRF7 | Protein tech，China | 22018-1-AP | 1:1000 | 1:1000 |  |  |
| METTL3 | Cell Signaling Technology, USA | 13008T | 1:1000 |  |  |  |
| Anti-CD3 | Biolegend，USA | 100203 |  |  |  | 1:100 |
| Anti-CD8 | Biolegend，USA | 100711 |  |  |  | 1:400 |
| Anti-CD25 | Biolegend，USA | 102007 |  |  |  | 1:100 |
| Anti-FOXP3 | Biolegend，USA | 126407 |  |  |  | 1:100 |
| Anti-CD11b | Biolegend，USA | 101236 |  |  |  | 1:400 |
| Anti-Ly-6G/Ly-6C | Biolegend，USA | 108443 |  |  |  | 1:400 |
| Anti-CD206 | Biolegend，USA | 141705 |  |  |  | 1:200 |
| Anti-F4/80 | Biolegend，USA | 123121 |  |  |  | 1:400 |
|  |  |  |  |  |  |  |

Abbreviations: WB: Western blot; IHC: Immunohistochemistry; IF: Immunofluorescence

**Table S2. Primers used in the experiment**s.

| Gene name | Primer sequence | | Application |
| --- | --- | --- | --- |
| E1 | F | 5’-GGGTGGTTGCAGTCAGTACA-3′ | qRT-PCR |
|  | R | 5’-GCAGCAATACACCAATCGCA-3′ |  |
| E2 | F | 5’-CACATGCGCCTAGAATGTGC-3′ | qRT-PCR |
|  | R | 5’-TTGGCCAAGTGCTGCCTAAT-3′ |  |
| E6 | F | 5’-CAGGAGCGACCCAGAAAGTT-3′ | qRT-PCR |
|  | R | 5’-GCAGTAACTGTTGCTTGCAGT-3′ |  |
| E7 | F | 5’-TGGACAAGCAGAACCGGAC-3′ | qRT-PCR |
|  | R | 5’-CAGATGGGGCACACAATTCCT-3′ |  |
| L1 | F | 5’-ACTTGCCTCCTGTCCCAGTA-3′ | qRT-PCR |
|  | R | 5’-CCACGACCTACCTCAACACC-3′ |  |
| L2 | F | 5’-CAAAACGTGCATCGGCTACC-3′ | qRT-PCR |
|  | R | 5’-AAGGGCCCACAGGATCTACT-3′ |  |
| ISG15 | F | 5’-GTGGACAAATGCGACGAACC-3′ | qRT-PCR |
|  | R | 5’-AGCATCTTCACCGTCAGGTC-3′ |  |
| OAS1 | F | 5’-GGAGACCCAAAGGGTTGGAG-3′ | qRT-PCR |
|  | R | 5’-GTGCAGGTCCAGTCCTCTTC-3′ |  |
| OAS2 | F | 5’-AAGCCAACGTGACATCCTCG-3′ | qRT-PCR |
|  | R | 5’-ACTCATGGCCTAGAGGTTGC-3′ |  |
| STAT3 | F | 5’-CATCCTGAAGCTGACCCAGG-3′ | qRT-PCR |
|  | R | 5’-TCCTCACATGGGGGAGGTAG-3′ |  |
| IRF3 | F | 5’-GACCTTCCATCGTAGGCCG-3′ | qRT-PCR |
|  | R | 5’-GGTTGGCAGGTCTGGCTTAT-3′ |  |
| IRF7 | F | 5’-GAGATCCATACCGAGGCAGC-3′ | qRT-PCR |
|  | R | 5’-TGCCCTCTCAGGAGCCAA-3′ |  |
| IFN-α | F | 5’-AACTCCCCTGATGAATGCGG-3′ | qRT-PCR |
|  | R | 5’-AGCAGGGGTGAGAGTCTTTG-3′ |  |
| IFN-β | F | 5’-GCTTGGATTCCTACAAAGAAGCA-3′  5’-ATAGATGGTCAATGCGGCGTC-3′ | qRT-PCR |
|  | R |  |  |
| IFN-γ | F | 5’-ACTGTCGCCAGCAGCTAAAA-3′  5’-GCTTAGGTTGGCTGCCTAGT-3′ | qRT-PCR |
|  | R |  |  |
| sh-YTHDF3  YTHDF3  YTHDF3 | shRNA  sgRNA-1  sgRNA-2 | CGGCATATTCGCTTAGAAA  tggtgtatttagtcaacctg  tgggtagctcctcgtaacag | sh-RNA  sgRNA  sgRNA |
| STAT3 | F | 5’-CAGAGTCTCAGACTGTCGCC-3′ | MeRIP-qPCR |
|  | R | 5’-ACATACTCCTGGCATTGCACT-3′ |  |

Abbreviations: F: Forward; R: Reverse; qRT-PCR: quantitative real-time PCR; RIP: RNA binding protein immunoprecipitation; MeRIP: Methylated RNA immunoprecipitation; si-RNA: Small interfering-RNA; sh-RNA: Short hairpin-RNA
