# Supplementary figures and images for: YTHDF3 suppresses interferon-stimulated gene (ISG)-dependent antitumor immunity and promotes HPV carcinogenesis in cervical cancer
Source: Cell Death Dis. 2025 Dec 26;17(1):60. doi: 10.1038/s41419-025-08188-6 (PMC12827990; doi:10.1038/s41419-025-08188-6)

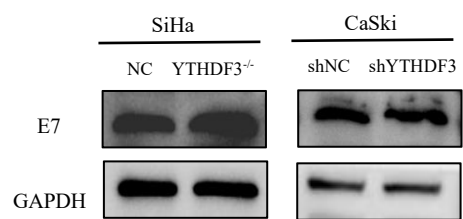

Supplement: Supplementary file 2 — Supplemental Figure1 [file 41419_2025_8188_MOESM2_ESM.pdf]
