# Supplementary material for: YTHDF3 suppresses interferon-stimulated gene (ISG)-dependent antitumor immunity and promotes HPV carcinogenesis in cervical cancer
Source: Cell Death Dis. 2025 Dec 26;17(1):60. doi: 10.1038/s41419-025-08188-6 (PMC12827990; doi:10.1038/s41419-025-08188-6)

Figure 1G

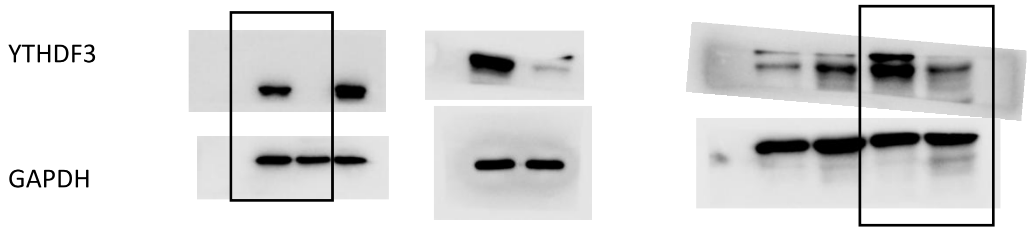

Figure 1J

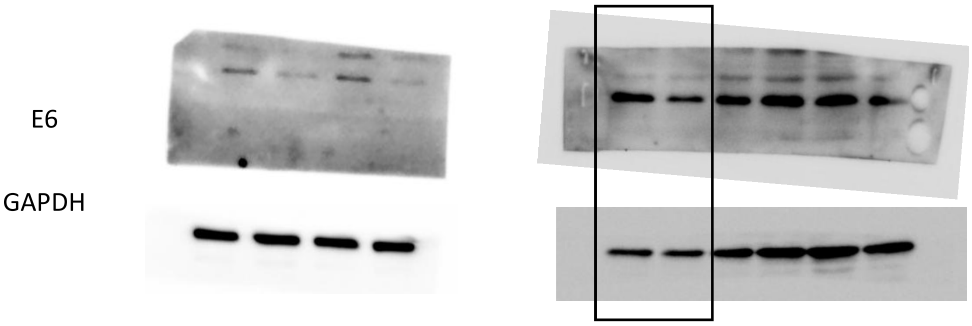

Figure 1L

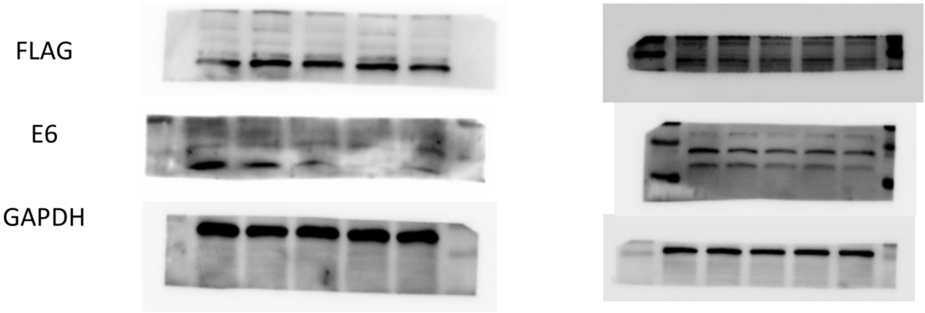

Figure 3G

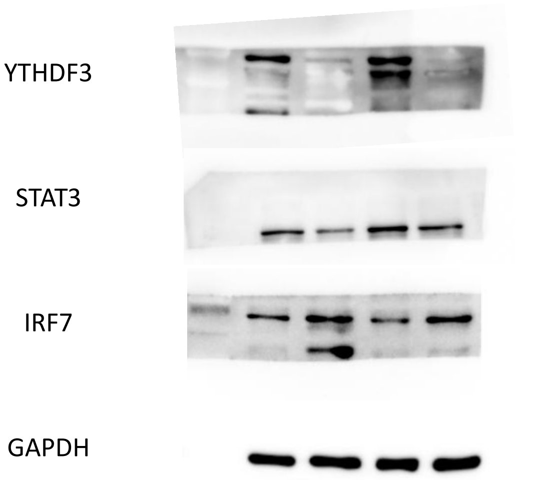

Figure 3H

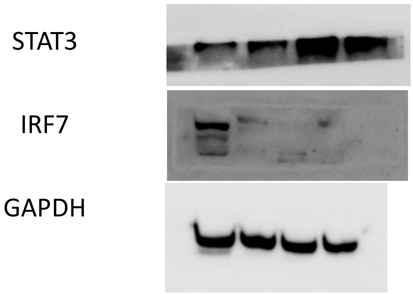

Figure 3I

METTL3

STAT3

GAPDH

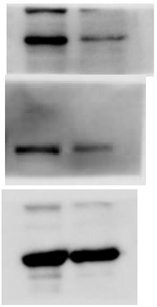

Figure 3L

STAT3

GAPDH

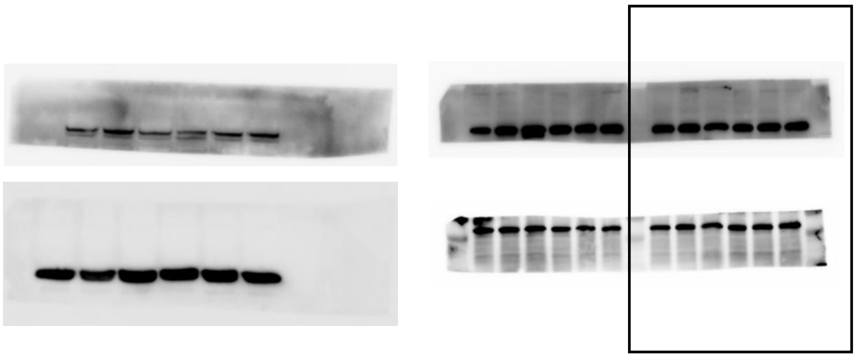

Figure 3N

STAT3

FLAG

GAPDH

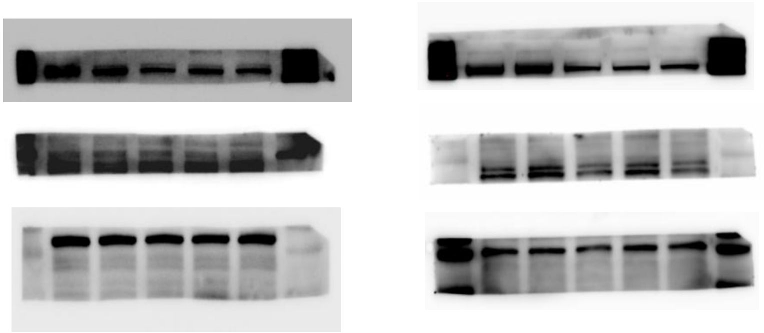

Figure 4C

STAT3

E6

GAPDH

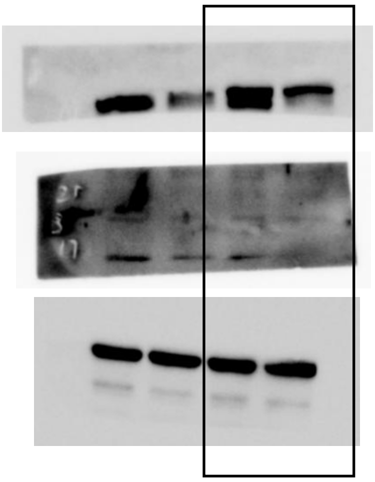

Figure 4D

STAT3

IRF7

GAPDH

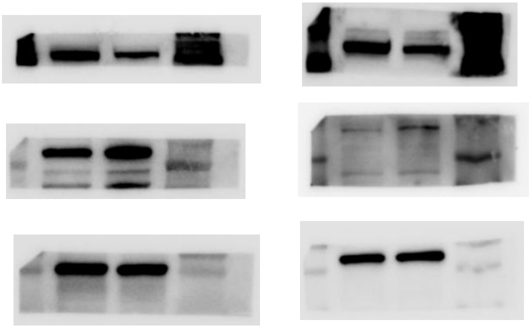

Figure 4E

STAT3

E6

GAPDH

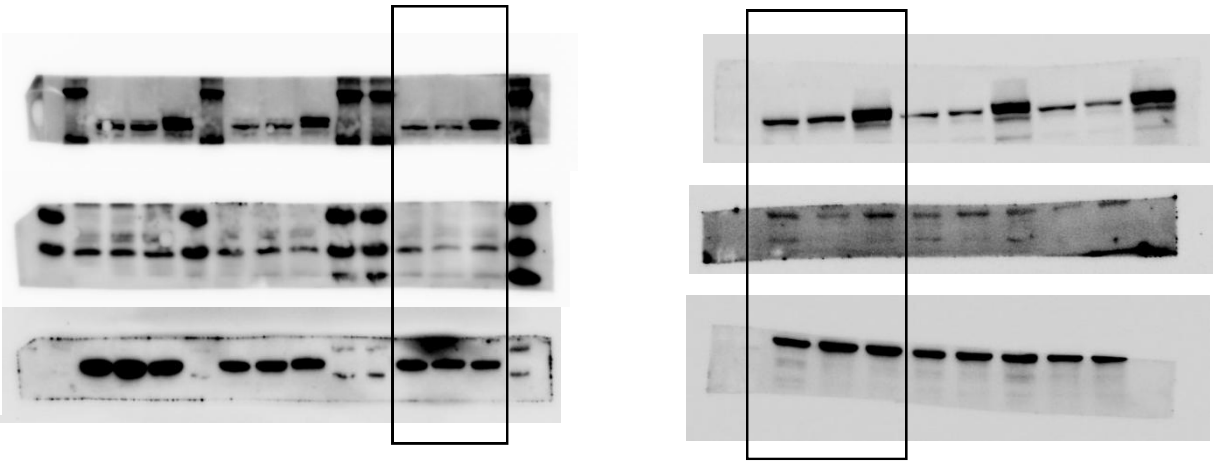

Figure 4F

STAT3

IRF7

GAPDH

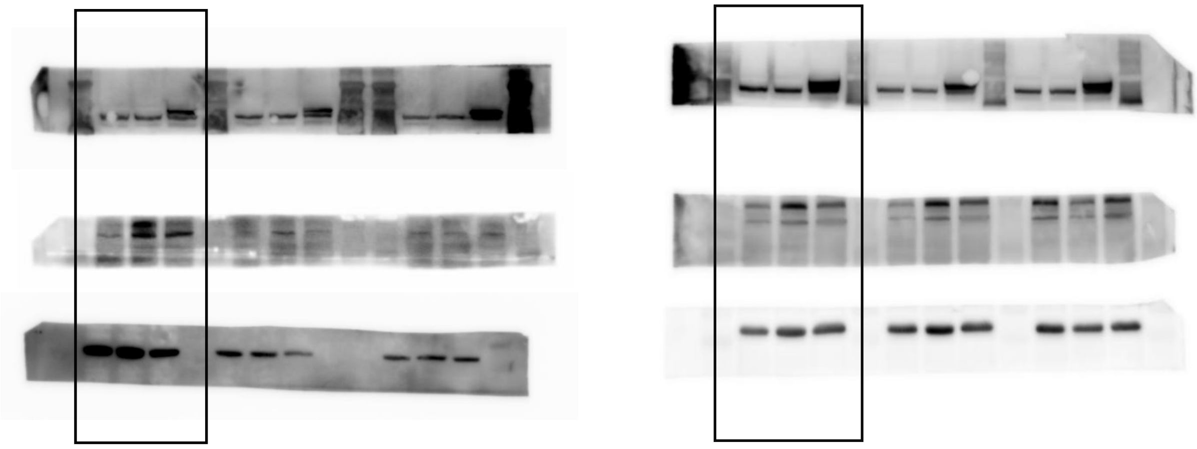

Supplementary Figure 1

E7

GAPDH

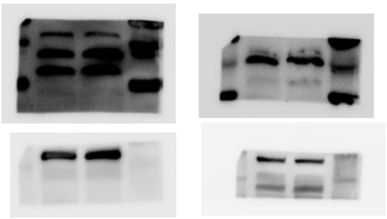

Supplement: Supplementary file 3 — Original blots [file 41419_2025_8188_MOESM3_ESM.pdf]
